# Associations between Apolipoprotein E Genotype, Diet, Body Mass Index, and Serum Lipids in Lithuanian Adult Population

**DOI:** 10.1371/journal.pone.0041525

**Published:** 2012-07-23

**Authors:** Janina Petkeviciene, Alina Smalinskiene, Dalia Ieva Luksiene, Kristina Jureniene, Vitalija Ramazauskiene, Jurate Klumbiene, Vaiva Lesauskaite

**Affiliations:** 1 Faculty of Public Health, Medical Academy, Lithuanian University of Health Sciences, Kaunas, Lithuania; 2 Institute of Cardiology, Medical Academy, Lithuanian University of Health Sciences, Kaunas, Lithuania; The University of Texas M. D. Anderson Cancer Center, United States of America

## Abstract

**Background:**

Apolipoprotein E (APOE) polymorphism is associated with lipid levels. Some studies have reported that blood lipid response to diet or obesity varies depending on *APOE* genotypes. The aim of this study was to assess the effect of *APOE* genotypes, the intake of saturated fatty acids (SFA), and obesity on serum lipid levels in Lithuanian adult population.

**Methodology/Principal Findings:**

A cross-sectional health survey was carried out in five municipalities of Lithuania. The random sample was obtained from lists of 25–64 year-old inhabitants registered at primary health care centres. The data from 996 subjects (416 men and 580 women) were analysed in this study. Two single-nucleotide polymorphisms (rs429358 and rs7412) were assessed using a real-time polymerase chain reaction. 24-hour recall and food frequency questionnaire were used for evaluation of dietary habits. Serum lipids were determined using enzymatic methods.

Men and women with the *APOE2* genotype had the lowest level of total cholesterol (TC) (p = 0.002 for men, and p = 0.02 for women) and low-density lipoprotein cholesterol (LDL-C) (p<0.001). Multivariate linear regression analysis showed that age, genotype *APOE2*, SFA intake, and body mass index (BMI) were significant determinants of TC and LDL-C level (with p values ranging from 0.043 to 0.001). Our data did not reveal any statistically significant interactions between *APOE* genotype and SFA intake or between *APOE* genotype and BMI regarding TC and LDL-C level (all p>0.05). However, the predictive power of the regression model for LDL-C improved when gene-BMI interaction and gene-BMI interaction plus gene-nutrient interaction were added (p = 0.04 and p = 0.032 for R^2^ change, respectively).

**Conclusions/Significance:**

*APOE* genotypes, SFA intake, and obesity were found to be associated with blood lipid levels in Lithuanian adult population. Analysis of gene-diet and gene-obesity interactions did not confirm that the effects of diet and obesity on TC and LDL-C level significantly depended on *APOE* genotype.

## Introduction

Dyslipidemia has been defined as one of the principal risk factors for the development of cardiovascular diseases (CVD) [1], [2]. Dietary habits such as saturated fatty acid (SFA) intake, obesity, and genetic factors are known to influence blood lipid profile [3]–[5]. Apolipoprotein E *(APOE)* plays an important role in lipid metabolism [6]. APOE mediates the uptake of lipoproteins through ligand-receptor interaction with the low-density lipoprotein (LDL) receptors [7]. APOE is polymorphic and has three major isoforms encoded by three alleles of chromosome 19 (*ε2*, *ε3* and *ε4*) [8]. APOE interactions with lipoprotein receptors depend on APOE isoform [9]. In most of the studied populations, the presence of the *APOE ε2* allele was associated with low levels of low-density lipoprotein cholesterol (LDL-C), whereas the *APOE ε4* allele was related to elevated levels of LDL-C [10]–[12].

Some studies have reported that diet may influence the effect of APOE polymorphism on blood lipid levels [13]–[15]. The data indicated that carriers of the *ε4* allele were more responsive to high fat and cholesterol intake than those without the *ε4* allele. Other studies failed to demonstrate that people with different *APOE* genotypes respond differently to dietary fat [16]–[18]. In addition to diet, a modulating effect of body mass index (BMI) on the association between the *APOE* genotype and blood lipids has been reported [19], [20].

High morbidity and mortality from CVD is a major health problem in Lithuania [21]. In 2009, the age-standardized mortality rate was 122.3 per 100 000 Lithuanian population aged 0–64 years, while the average rate in the European Union was 45.6 per 100 000 population of the same age [22]. Disparities in CVD mortality can be linked to a number of complex factors, such as economic, social, lifestyle, and genetic factors. In Lithuania, the transition period from a centralized communist to a market-oriented economy was characterized by some positive changes in the food habits of the population. The use of vegetable fats and the frequency of consumption of fresh vegetables increased. Conversely, the use of animal fats declined. However, epidemiological studies have demonstrated that diet-related CVD risk factors including dyslipidemia and obesity are still highly prevalent in the country [23], [24]. Furthermore, there is a lack of data regarding the role of genetic factors and gene-environmental interactions for CVD risk in Lithuania. Such data can help to assess the risk of CVD more accurately and to define the most vulnerable groups of people who would benefit from interventions.

The aim of this study was to assess the relationships of *APOE* genotypes, dietary intake, and body mass index with serum lipid levels in Lithuanian adult population.

## Materials and Methods

### Ethics Statement

The study protocol was approved by the Lithuanian Bioethics Committee. Written informed consent for the participation in the study was obtained from all participants.

### Study design and sample

The cross-sectional health survey was carried out in five municipalities, with populations ranging from 20 to 45 thousands, randomly selected from the northern, southern, eastern, western and central parts of Lithuania. The random sample was obtained from lists of 25–64 year-old inhabitants registered at the primary health care centres of the included municipalities. In Lithuania, the majority of the population is registered with a primary health care institution [21]. In 2007, health examinations were conducted for 1739 participants (58% of the eligible sample). From these, 1035 randomly selected individuals (429 men and 606 women) had their *APOE* genotypes determined. Those individuals did not differ from other participants of health examination with respect to basic demographic characteristics, lipid levels, and BMI. We excluded 34 subjects with the rare *APOE ε2*/*4* genotype and 5 subjects taking lipid-lowering medications from our analysis. Finally, data of 996 subjects (416 men and 580 women) were analysed in this study.

### *APOE* genotyping

For DNA extraction, blood samples were collected from each individual in ethylenediaminetetraacetic (EDTA) tubes during their health examination. DNA was extracted from peripheral blood leukocytes using a reagent kit (NucleoSpin Blood L Kit; Macherey & Nagel, Düren, Germany).

Two single-nucleotide polymorphisms (SNPs) (rs429358 and rs7412) were assessed using TaqMan assays (Applied Biosystems, Foster City, CA, USA). We used the Applied Biosystems 7900HT Real-Time Polymerase Chain Reaction System for detecting the SNPs. The cycling program started with heating at 95°C for 10 min, followed by 40 cycles (at 95°C for 15 s and at 60°C for 1 min). Allelic discrimination was carried out using the software of Applied Biosystems. The allele *APOE ε2* has a cytosine-to-thymine base-pair substitution in rs7412, while in *APOE ε4*, a thymine is replaced by a cytosine in rs429358.

Three genotype groups were analysed in this study: *APOE2* (carriers of the *ε 2/2* and the *ε 2/3* genotype), *APOE3* (carriers of the *ε 3/3*genotype), and *APOE4* (carriers of the *ε 3/4* and the *ε 4/4* genotype).

### Laboratory analyses and anthropometric measurements

Blood samples for lipid levels measurements were taken in the morning after at least 12 hours of fasting. TC, LDL-C, HDL-C, and TG levels were determined using an automatic analyser under conventional enzymatic methodology. All laboratory analyses were made in the same certified laboratory. Quality control measures were followed for the estimation of lipid concentrations.

The height of participants (without shoes) was measured with the accuracy of one centimeter, using a stadiometer. The body weight of participants, wearing light indoor clothing and no shoes, was measured with the accuracy of 0.1 kg, using standardized medical scales. BMI was calculated as weight divided by height squared (kg/m^2^). Obesity was defined as BMI≥30 kg/m^2^.

### Dietary assessment

A 24-hour dietary recall and food frequency questionnaire were used for the assessment of dietary intake [25]. The questionnaire included questions about food habits related to meat and milk consumption as well as the type of fat used in cooking and spreading on bread. Trained dietary interviewers collected the data. Food models, a validated picture book, and household measures were used to quantify food portions sizes. Nutrient values of food were calculated using the Lithuanian Food Composition Tables [26], [27].

### Statistical analysis

Statistical analysis was performed using the SPSS software package, version 19.0 for Windows. Categorical variables were expressed as percentages and tested by the χ^2^ test. Hardy-Weinberg equilibrium was also assessed using the χ^2^ test. The normality of the distribution of continuous variables was tested by the Kolmogorov - Smirnov test. Only distribution of triglyceride levels was skewed, and this variable was logarithmically transformed to improve normality for statistical testing. Estimated means were subsequently back-transformed for presentation in the tables. Analysis of variance with Bonferroni multiple comparison tests were used to compare the mean values of continuous variables across groups.

The dietary variable included in the analysis was the percent of total energy intake (E%) from SFA, because only this nutrient was associated with lipid levels. Multiple linear regression models were applied to test the effects of *APOE* genotypes, SFA intake, and BMI on lipid levels, controlling for sex and age. Separate models included the main effect and interaction terms (gene-nutrient or/and gene-BMI interactions). Genotypes were included as dummy variables. In order to test whether the interaction terms added any significant predictive power to the regression model, change in R^2^ and the significance of R^2^ change were calculated. A p-value less than 0.05 was considered to be statistically significant.

## Results

The prevalence of *APOE* genotypes and alleles in the study population is presented in Table 1. The *APOE* allele frequencies were 0.10 for *APOE ε2*, 0.78 for *APOE ε3*, and 0.12 for *APOE ε4*. We did not observe any significant differences in the frequencies of genotypes or alleles between men and women. Furthermore, frequencies of genotypes in men and women did not differ significantly from those predicted by the Hardy-Weinberg equilibrium (χ^2^ = 4.91; p = 0.178 for men, and χ^2^ = 4.88; p = 0.181 for women).

**Table 1:** Prevalence (%) of apolipoprotein E genotypes and frequency of alleles in the study population.

	Men (n = 429)		Women (n = 606)		Total (n = 1035)	
	N	%	n	%	n	%
Genotype						
*ε 2/2*	2	0.5	2	0.3	4	0.4
*ε 3/2*	77	17.9	95	15.7	172	16.6
*ε 3/3*	273	63.6	362	59.7	635	61.3
*ε 4/2*	13	3.0	21	3.5	34	3.3
*ε 4/3*	62	14.5	117	19.3	179	17.3
*ε 4/4*	2	0.5	9	1.5	11	1.1
Allele						
*ε2*	0.11		0.10		0.10	
*ε3*	0.80		0.77		0.78	
*ε4*	0.09		0.13		0.11	

The characteristics of study subjects according to *APOE* genotypes are given in Table 2 and Table 3. Mean values of age, intake of SFA, and BMI, as well as the prevalence of obesity, were similar in groups with different genotypes. An association between *APOE* genotypes and the lipid levels was observed. Men and women with *APOE2* genotype had the lowest level of TC and LDL-C. Mean values of TC and LDL-C were highest in the *APOE4* genotype group; however, they did not differ significantly from mean values of lipids of *APOE3* genotype carriers. No statistically significant effect of *APOE* genotypes on the level of HDL-C and TG was observed.

**Table 2:** Characteristics of the population (means and standard deviations (SD) or percentage) according to apolipoprotein E genotype (men).

Characteristics	Apolipoprotein E genotype			
	*APOE2*	*APOE3*	*APOE4*	p ANOVA
Age (years)	46.5 (10.5)	48.1 (10.8)	48.1 (9.2)	0.462
TC (mmol/l)	4.99 (1.10)	5.37* (1.04)	5.56* (0.97)	0.002
LDL-C (mmol/l)	2.97 (0.82	3.49* (1.02)	3.61* (0.93)	<0.001
HDL-C (mmol/l)	1.23 (0.47)	1.30 (0.40)	1.33 (0.60)	0.393
TG^a^ (mmol/l)	1.71 (0.73)	1.59 (0.90)	1.68 (0.66)	0.052
BMI (kg/m^2^)	27.4 (5.0)	27.6 (5.1)	27.6 (4.5)	0.943
Obesity (%)	24.4	27.3	23.8	0.812
SFA (E %)	14.1 (4.6)	14.9 (5.2)	14.4 (4.0)	0.943

* p<0.05 compared to *APOE2* (Post hoc analysis with Bonferroni corrections).

a Triglycerides were logarithmically transformed before analysis, and estimated means were subsequently back-transformed for presentation in the Tables.

Abbreviations: *APOE* – apolipoprotein E; TC – total cholesterol; LDL-C – low-density lipoprotein cholesterol; HDL-C – high-density lipoprotein cholesterol; TG – triglycerides; SFA – saturated fatty acids; E% – percentage of total energy intake.

**Table 3:** Characteristics of the population (means and standard deviations (SD) or percentage) according to apolipoprotein E genotype (women).

Characteristics	Apolipoprotein E genotype			
	*APOE2*	*APOE3*	*APOE4*	p ANOVA
Age (years)	46.0 (10.1)	46.6 (10.8)	45.2 (11.6)	0.427
TC (mmol/l)	5.02 (1.17)	5.35* (0.98)	5.35* (1.14)	0.02
LDL-C (mmol/l)	2.72 (1.01)	3.32* (0.91)	3.33* (0.99)	<0.001
HDL-C (mmol/l)	1.48 (0.43)	1.41 (0.34)	1.44 (0.42)	0.262
TG^a^ (mmol/l)	1.44 (0.63)	1.47 (0.61)	1.44 (0.51)	0.861
BMI (kg/m^2^)	27.2 (5.8)	28.2 (7.2)	28.3 (6.3)	0.372
Obesity (%)	27.1	30.7	36.5	0.270
SFA (E %)	13.7 (4.44)	14.6 (4.7)	14.3 (5.2)	0.372

* p<0.05 compared to *APOE2* (Post hoc analysis with Bonferroni corrections).

a Triglycerides were logarithmically transformed before analysis, and estimated means were subsequently back-transformed for presentation in the Tables.

Abbreviations: *APOE* – apolipoprotein E; TC – total cholesterol; LDL-C – low-density lipoprotein cholesterol; HDL-C – high-density lipoprotein cholesterol; TG – triglycerides; SFA – saturated fatty acids; E% – percentage of total energy intake.

The prevalence of obesity in the study population was 28.9% (95% CI: 26.1–31.7). Overweight and obesity were associated with an atherogenic blood lipid profile (Table 4). Overweight and obese subjects had higher levels of TC, LDL-C, and TG and lower levels of HDL-C than people with normal BMI did. The diet of the study population was very high in SFA. The average share of SFA in total energy intake was 14.7 (4.9) E% in men and 14.3 (4.8) E% in women (p>0.05). High intake of SFA was related to higher levels of TC, LDL-C, and TG (Table 4).

**Table 4:** Mean serum lipid levels (mmol/l), according to body mass index and saturated fatty acid intake.

Lipids	Body mass index				Saturated fatty acid intake (E%)		
	<25	25–29	≥30	p^a^	≤14.2	>14.2	p^b^
TC	5.07	5.41*	5.48*	<0.001	5.20	5.40	0.005
SD	0.94	1.07	1.10		1.04	1.08	
LDL-C	2.99	3.43*	3.52*	<0.001	3.22	3.36	0.034
SD	0.89	0.98	1.00		1.00	0.98	
HDL-C	1.53	1.36*	1.23* #	<0.001	1.39	1.37	0.476
SD	0.44	0.41	0.32		0.40	0.41	
TG	1.33	1.53*	1.73* #	<0.001	1.46	1.54	0.042
SD	0.48	0.64	0.92		0.66	0.75	

Estimates are age-adjusted. Triglycerides were logarithmically transformed before analysis, and estimated means were subsequently back-transformed for presentation in the Tables.

a p calculated using ANOVA;

b p calculated using Student t test.

* p<0.001 compared to BMI<25;

# p<0.001 compared to BMI 25–29 (Post hoc analysis with Bonferroni corrections).

Abbreviations: SD – standard deviation; TC – total cholesterol; LDL-C – low-density lipoprotein cholesterol; HDL-C – high-density lipoprotein cholesterol; TG – triglycerides; BMI – body mass index; SFA – saturated fatty acids; E% – percentage of total energy intake.

The regression model showed that age, genotype *APOE2*, SFA intake, and BMI were the significant determinants of TC and LDL-C (Model 1) (Table 5 and Table 6). The presence of *APOE2* genotype contributed negatively to lipid levels. After including the gene-nutrient interaction into the model, the contribution of *APOE4* genotype to both TC and LDL-C reached statistical significance (Model 2). The model with gene-BMI interaction (Model 3), as well as gene-nutrient interaction plus gene-BMI interaction (Model 4), showed a significant contribution of age, SFA intake, and BMI to lipid levels. We did not find any statistically significant interaction between *APOE* genotypes and SFA intake or between *APOE* genotypes and BMI in any of the regression models. With the addition of the terms of interaction to the regression model predicting TC, R^2^ increased only slightly, and the change in R^2^ was not statistically significant (Table 5). Hence, the interactions did not add any significant predictive power to the regression model. Meanwhile, the inclusion of gene-BMI interaction and gene-BMI interaction plus gene-nutrient interaction statistically significant improved the predictive power of the regression model predicting LDL-C, although the change in R^2^ was small (Table 6).

**Table 5:** Association of apolipoprotein E genotype, SFA intake, and BMI with total cholesterol.

Models	Model 1	Model 2	Model 3	Model 4
	β (SE)	β (SE)	β (SE)	β (SE)
Women vs men	−0.062 (0.070)	−0.062 (0.070)	−0.063 (0.070)	−0.063 (0.070)
P	0.380	0.378	0.369	0.367
Age (years)	0.026 (0.003)	0.026 (0.003)	0.025 (0.003)	0.025 (0.003)
P	<0.001	<0.001	<0.001	<0.001
Dummy APOE2	−0.253 (0.095)	0.111 (0.304)	0.241 (0.487)	0.589 (0.561)
P	0.008	0.715	0.620	0.294
Dummy APOE4	0.152 (0.090)	0.582 (0.282)	−0.467 (0.452)	−0.049 (0.515)
P	0.091	0.039	0.301	0.924
SFA	0.021 (0.007)	0.030 (0.009)	0.020 (0.007)	0.029 (0.009)
P	0.004	0.001	0.005	0.001
BMI	0.013 (0.006)	0.013 (0.006)	0.020 (0.009)	0.020 (0.009)
P	0.028	0.025	0.016	0.017
APOE2*SFA		−0.026 (0.021)		−0.025 (0.021)
P		0.212		0.220
APOE4*SFA		−0.030 (0.019)		−0.031 (0.018)
P		0.108		0.093
APOE2*BMI			−0.018 (0.018)	−0.018 (0.018)
P			0.293	0.301
APOE4*BMI			0.022 (0.016)	0.023 (0.016)
P			0.171	0.151
R^2^	0.117	0.121	0.121	0.125
ΔR^2^		0.004	0.004	0.008
p^a^		0.180	0.144	0.112

Regression coefficients are expressed in kg/m^2^ for BMI and in mmol/l for total cholesterol.

Model 1 – without interactions; Model 2 – with gene-SFA intake interaction; Model 3 – with gene-BMI interaction; Model 4 – with gene-SFA intake interaction and gene-BMI interaction.

ΔR^2^ - R^2^ change compared to Model 1; p^a^ - p for R^2^change.

Abbreviations: *APOE* – apolipoprotein E; BMI – body mass index; SFA – saturated fatty acids; SE – standard error.

**Table 6:** Association of apolipoprotein E genotype, SFA intake, and BMI with low-density lipoprotein cholesterol.

Models	Model 1	Model 2	Model 3	Model 4
	β (SE)	β (SE)	β (SE)	β (SE)
Women vs men	−0.197 (0.065)	−0.197 (0.064)	−0.198 (0.064)	−0.198 (0.064)
P	0.002	0.002	0.002	0.002
Age (years)	0.021 (0.003)	0.020 (0.003)	0.019 (0.003)	0.019 (0.003)
P	<0.001	<0.001	<0.001	<0.001
Dummy APOE2	−0.457 (0.087)	−0.172 (0.280)	0.213 (0.446)	0.479 (0.514)
P	<0.001	0.537	0.632	0.351
Dummy APOE4	0.117 (0.083)	0.568 (0.259)	−0.543 (0.414)	−0.106 (0.472)
P	0.159	0.028	0.190	0.823
SFA	0.013 (0.007)	0.022 (0.008)	0.012 (0.007)	0.021 (0.008)
P	0.043	0.006	0.05	0.007
BMI	0.023 (0.005)	0.023 (0.005)	0.032 (0.008)	0.032 (0.008)
P	<0.001	<0.001	<0.001	<0.001
APOE2*SFA		−0.020 (0.019)		−0.019 (0.01)
P		0.291		0.308
APOE4*SFA		−0.031 (0.017)		−0.033 (0.017)
P		0.066		0.054
APOE2*BMI			−0.025 (0.016)	−0.025 (0.016)
P			0.121	0.124
APOE4*BMI			0.023 (0.015)	0.024 (0.015)
P			0.112	0.096
R^2^	0.151	0.154	0.157	0.161
ΔR^2^		0.004	0.007	0.011
p^a^		0.150	0.040	0.032

Regression coefficients are expressed in kg/m^2^ for BMI and in mmol/l for low-density lipoprotein cholesterol.

Model 1 – without interactions; Model 2 – with gene-SFA intake interaction; Model 3 – with gene-BMI interaction; Model 4 – with gene-SFA intake interaction and gene-BMI interaction.

ΔR^2^ - R^2^ change compared to Model 1; p^a^ - p for R^2^ change.

Abbreviations: *APOE* – apolipoprotein E; BMI – body mass index; SFA – saturated fatty acids; SE – standard error.

Next, we assessed the association of SFA intake and BMI with lipid levels using multiple linear regression analysis stratified by genotype. A statistically significant contribution of SFA intake to TC and LDL-C levels was only found in *APOE3* genotype carriers (β = 0.029, p<0.001 for TC and β = 0.022, p = 0.006 for LDL-C). BMI was associated with lipid levels among individuals with the *APOE3* and *APOE4* genotype. The effect of BMI was stronger in *APOE4* genotype carriers (β = 0.041, p = 0.003 for TC and β = 0.054, p<0.001 for LDL-C) (data not shown).

We used multivariate regression models to test the effects of *APOE* genotypes, SFA intake, and BMI on HDL-C and TG levels. Only BMI contributed negatively to HDL-C level (β = −0.020, p<0.001) and positively - to TG level (β = 0.025, p<0.001). Neither *APOE* genotypes nor SFA intake were significant determinants of HDL-C and TG levels. Furthermore, we did not find any statistically significant gene-nutrient and gene-BMI interactions in the regression models predicting HDL-C and TG levels (data not shown).

## Discussion

To date, this is the first study that assessed the frequencies of the *APOE* allele, the association of the *APOE* genotypes with lipid levels, and the effect of the interaction between *APOE* genotypes, SFA intake, and obesity on lipid levels in the adult Lithuanian population. The frequencies of the *APOE* allele in our population were comparable with previously reported data for other European populations [28]–[31].

Our study demonstrated that the *APOE2* genotype was associated with the lowest level of TC and LDL-C, confirming findings from other studies [10]–[12], [20], [29], [30]. Some observations proved that subjects with *APOE2* genotype had higher levels of TG than those with the *APOE3* genotype [18], [32]. APOE2 was found to bind poorly to lipoprotein receptors leading to the accumulation of chylomicron and very low-density lipoprotein remnants [7]–[9]. In our study, the concentration of TG tended to be higher in men with the *APOE2* genotype compared to those with the *APOE3* genotype, but no such differences were detected in women. Previous studies demonstrated that the effects of *APOE* genotype on lipid levels differ by gender [32], [33]. The reasons for those differences are not studied well; however, some authors stated that they might be related to the influence of sex hormones [32].

In our study, high SFA intake was associated with increased levels of TC, LDL-C, and TG. The average share of SFA in total energy intake of Lithuanians (14.5 E%) was higher than that recommended for CVD prevention (less than 10 E%) [34]. Positive changes in nutrition habits of Lithuanian population, which occurred over two decades of post-communist transition period (the increase in the use of vegetable oil in cooking and the decrease in butter and high-fat milk consumption), improved the quality of fat intake and contributed to the decline in mean TC and LDL-C levels [25], [35]. However, the increased purchasing power of the inhabitants stimulated a rise in the consumption of meat and meat products [36]. An increase in energy intake and a decline in physical activity resulted in a high prevalence of obesity. Since 1990s, the proportion of obese men almost doubled in Lithuania [37]. Our study demonstrated that obese people had the atherogenic lipid profile.

It is well known that individuals vary widely in their response to any diet. This variation in response to dietary intake could be associated with genetic polymorphisms [38], [39]. Among environmental factors, diet may be a major factor involved in the modulation of genetic susceptibility to some diseases. A genetic risk factor may lead to disorder only under certain environmental conditions - for example, unhealthy diet [38], [39]. Although the intake of saturated fat raises the mean TC level of a population leading to an increased risk of CVD, not all individuals within the population are susceptible. Several studies have reported that subjects with *APOE4* genotype are more likely to respond to high SFA intake with increased levels of LDL-C than those having another *APOE* genotype [13], [14], [40]. Moreover, the association between *APOE4* genotype and LDL-C was stronger in populations consuming high SFA diet than in those with low SFA intake. In Costa Rica, where the diet of the population was low in SFA, higher SFA intake was associated with increased levels of very low-density lipoprotein cholesterol, lower HDL-C levels, and smaller LDL particles in *APOE2* genotype carriers, while the opposite effect was found in *APOE4* genotype carriers [41]. The authors concluded that individuals with *APOE2* genotype might have a greater risk of CVD when exposed to diets high in SFA. Kofler et al. reported that the association between TG levels and BMI was the also strongest among *APOE2* carriers [20].

Our study did not reveal any significant effect of the interaction between *APOE* genotype and SFA intake or between *APOE* genotype and BMI on blood lipid levels. However, the predictive power of the regression model for LDL-C improved when gene-BMI interaction and gene-BMI interaction plus gene-nutrient interaction were added. Several reasons explaining why statistical tests for gene-diet and gene-obesity interactions were not significant can be proposed. The sample size of our study was relatively small, affecting its ability and power to detect the effect of such interactions on lipid levels. A diet high in saturated fat was typical for the majority of the study population, and thus the possibility to evaluate the effect of lower SFA intake on lipid levels with sufficient power was limited. Furthermore, the cross-sectional study design did not allow us to consider all possible genetic and environmental confounders that may influence lipid levels. The development of dyslipidemias depends on the genetic diversity of individuals, the complexity of dietary habits, and a variety of other heath behaviours. Further research, particularly intervention studies, will be necessary to assess the variability in response to diet in relation to genetic factors.

## References

[1] Sharrett AR, Ballantyne CM, Coady SA, Heiss G, Sorlie PD (2001). Coronary heart disease prediction from lipoprotein cholesterol levels, triglycerides, lipoprotein(a), apolipoproteins A-I and B, and HDL density subfractions: The Atherosclerosis Risk in Communities (ARIC) Study.. Circulation.

[2] Emerging Risk Factors Collaboration (2009). Major lipids, apolipoproteins, and risk of vascular disease.. JAMA.

[3] Mensink RP, Zock PL, Kester AD, Katan MB (2003). Effects of dietary fatty acids and carbohydrates on the ratio of serum total to HDL cholesterol and on serum lipids and apolipoproteins: a metaanalysis of 60 controlled trials.. Am J Clin Nutr.

[4] Franssen R, Monajemi H, Stroes ES, Kastelein JJ (2008). Obesity and dyslipidemia.. Endocrinol Metab Clin North Am.

[5] Hegele RA (2009). Plasma lipoproteins: genetic influences and clinical implications.. Nat Rev Genet.

[6] Utermann G, Langenbeck U, Beisiegel U, Weber W (1980). Genetics of the apolipoprotein E system in man.. Am J Hum Genet.

[7] Mahley RW (1988). Apolipoprotein E: cholesterol transport protein with expanding role in cell biology.. Science.

[8] Weisgraber KH, Rall SC, Mahley RW (1981). Human E apoprotein heterogeneity. Cysteine-arginine interchanges in the amino acid sequence of the apo-E isoforms.. J Biol Chem.

[9] Weisgraber KH (1994). Apolipoprotein E: structure-function relationships.. Adv Protein Chem.

[10] Frikke-Schmidt R, Nordestgaard BG, Agerholm-Larsen B, Schnohr P, Tybjaerg-Hansen A (2000). Context-dependent and invariant associations between lipids, lipoproteins, and apolipoproteins and apolipoprotein E genotype.. J Lipid Res.

[11] Bennet AM, Di Angelantonio E, Ye Z, Wensley F, Dahlin A (2007). Association of apolipoprotein E genotypes with lipid levels and coronary risk.. JAMA.

[12] Ward H, Mitrou PN, Bowman R, Luben R, Wareham NJ (2009). APOE genotype, lipids, and coronary heart disease risk.. Arch Intern Med.

[13] Sarkkinen E, Korhonen M, Erkkilä A, Ebeling T, Uusitupa M (1998). Effect of apolipoprotein E polymorphism on serum lipid response to the separate modification of dietary.. Am J Clin Nutr.

[14] Weggemans RM, Zock PL, Ordovas JM, Pedro-Botet J, Martijn B (2001). Apoprotein E genotype and the response of serum cholesterol to dietary fat, cholesterol and cafestol.. Atherosclerosis.

[15] de Andrade FM, Bulhoes AC, Maluf SW, Schuch JB, Voigt F (2010). The influence of nutrigenetics on the lipid profile: interaction between genes and dietary habits.. Biochem Genet.

[16] Lefevre M, Ginsberg HN, Kris-Etherton PM, Elmer PJ, Stewart PW (1997). ApoE genotype does not predict lipid response to changes in dietary saturated fatty acids in a heterogeneous normolipidemic population. The DELTA Research Group. Dietary Effects on Lipoproteins and Thrombogenic Activity.. Arterioscler Thromb Vasc Biol.

[17] Salminen M, Lehtimäki T, Fan YM, Vahlberg T, Kivelä SL (2006). Apolipoprotein E polymorphism and changes in serum lipids during a family-based counselling intervention.. Public Health Nutr.

[18] Wu K, Bowman R, Welch AA, Luben RN, Wareham N (2007). Apolipoprotein E polymorphisms, dietary fat and fibre, and serum lipids: the EPIC Norfolk study.. Eur Heart J.

[19] Marques-Vidal P, Bongard V, Ruidavets JB, Fauvel J, Hanaire-Broutin H (2003). Obesity and alcohol modulate the effect of apolipoprotein E polymorphism on lipids and insulin.. Obes Res.

[20] Kofler BM, Miles EA, Curtis P, Armah CK, Tricon S (2012). Apolipoprotein E genotype and the cardiovascular disease risk phenotype: impact of sex and adiposity (the FINGEN study).. Atherosclerosis.

[21] Health Statistics of Lithuania (2009). http://www.lsic.lt/data/la2009.pdf.

[22] European Health for All Database (HFA-DB) http://www.euro.who.int/hfadb.

[23] Reklaitiene R, Margeviciene L, Tamosiunas A, Domarkiene S, Buivydaite K (2001). Trends in prevalence of dyslipidaemias and risk of coronary heart disease among 25–64 aged Kaunas population.. Medicina.

[24] Grabauskas V, Klumbienė J, Petkeviciene J, Petrauskiene A, Tamosiunas A (2008). Risk factors for non-communicable diseases in Lithuania rural population: CINDI survey 2007.. Medicina.

[25] Ramazauskiene V, Petkeviciene J, Klumbiene J, Kriaucioniene V, Sakytė E (2011). Diet and serum lipids: changes over socio-economic transition period in Lithuanian rural population.. BMC Public Health.

[26] Suciliene S, Abaravicius A, Kadziauskiene K, Barzda A, Bartkeviciute R (2002). Maisto produktų sudėtis.

[27] Barzda A, Olechnovic M, Barkeviciute R, Abaravicius A, Stukas R (2005). Patiekalų sudėtis, maistinė ir energetinė vertė.

[28] Schiele F, De Bacquer D, Vincent-Viry M, Beisiegel U, Ehnholm C (2000). Apolipoprotein E serum concentration and polymorphism in six European countries: the ApoEurope Project.. Atherosclerosis.

[29] Bednarska-Makaruk M, Broda G, Kurjata P, Rodo M, Roszczynko M (2001). Apolipoprotein E genotype, lipid levels and coronary heart disease in a Polish population group.. Eur J Epidemiol.

[30] Hubacek JA, Pitha J, Adamkova V, Skodova Z, Lanska V (2003). Apolipoprotein E and apolipoprotein CI polymorphisms in the Czech population: almost complete linkage disequilibrium of the less frequent alleles of both polymorphisms.. Physiol Res.

[31] Singh PP, Singh M, Mastana SS (2006). APOE distribution in world populations with new data from India and the UK.. Ann Hum Biol.

[32] Frikke-Schmidt R, Tybjaerg-Hansen A, Steffensen R, Jensen G, Nordestgaard BG (2000). Apolipoprotein E genotype: epsilon32 women are protected while epsilon43 and epsilon44 men are susceptible to ischemic heart disease: the Copenhagen City Heart Study.. J Am Coll Cardiol.

[33] Reilly SL, Ferrell RE, Sing CF (1994). The gender-specific apolipoprotein E genotype influence on the distribution of plasma lipids and apolipoproteins in the population of Rochester, MN. III. Correlations and covariances.. Am J Hum Genet.

[34] WHO (World Health Organization) (2003). Diet, nutrition, and the prevention of chronic disease - Report of a joint WHO/FAO expert consultation..

[35] Kriaucioniene V, Klumbiene J, Petkeviciene J, Sakyte E (2012). Time trends in social differences in nutrition habits of a Lithuanian population: 1994–2010.. BMC Public Health.

[36] Prattalla R, Helakorpi S, Sipila N, Sippola R, Saaksjarvi K (2011). Social determinants of health behaviours: Finbalt Health Monitor 1998–2008.

[37] Grabauskas V, Klumbiene J, Petkeviciene J, Sakyte E, Kriaucioniene V (2011). Health behaviour among Lithuanian adult population, 2010.

[38] Ordovas JM (2006). Genetic interactions with diet influence the risk of cardiovascular disease.. Am J Clin Nutr.

[39] Corella D, Ordovas JM (2009). Nutrigenomics in cardiovascular medicine.. Circ Cardiovasc Genet.

[40] Tikkanen MJ (1997). Apolipoprotein E polymorphism and plasma cholesterol response to dietary change.. World Rev Nutr Diet.

[41] Campos H, D'Agostino M, Ordovás JM (2001). Gene-diet interactions and plasma lipoproteins: role of apolipoprotein E and habitual saturated fat intake.. Genet Epidemiol.

